# Prediction of high-grade cervical precancerous abnormalities: The role of personal factors, vaginal microflora, sexually transmitted infections, and high-risk human papillomavirus

**DOI:** 10.1371/journal.pone.0313004

**Published:** 2024-11-11

**Authors:** Olga Plisko, Jana Zodzika, Irina Jermakova, Kristine Pcolkina, Amanda Prusakevica, Inta Liepniece-Karele, Marta Zarina, Jelena Storozenko, Dace Rezeberga

**Affiliations:** 1 Department of Obstetrics and Gynecology, Riga Stradins University, Riga, Latvia; 2 Gynecological Clinic, Riga East Clinical University Hospital, Riga, Latvia; 3 Faculty of Residency, Riga Stradins University, Riga, Latvia; 4 Department of Pathology, Riga Stradins University, Riga, Latvia; 5 Pathology Center, Riga East Clinical University Hospital, Riga, Latvia; 6 Department of Infectology, Riga Stradins University, Riga, Latvia; 7 Central Laboratory, Riga, Latvia; Wanye State University, UNITED STATES OF AMERICA

## Abstract

High-risk human papillomavirus infection (HR-HPV) is necessary but not the only factor needed to develop cervical cancer. It is essential to estimate cervical cancer development risk in the population of high-risk HPV-positive women and to avoid unnecessary examinations and treatment in low-risk individuals. The study aimed to identify associations between different personal factors, vaginal microflora, sexually transmitted, high-risk HPV infection, and various degrees of cervical precancerous lesions. A study was performed in 2016–2020. The study group consisted of 112 patients with abnormal cervical cytology results referred for colposcopic examination. 120 women who came for a routine gynecological check-up were included in the control group. Material from the cervix and upper vaginal fornix was taken for pH measurement, wet mount microscopy, testing the six most common high-risk HPV DNA types (16/18, 31, 33, 45, 58), HPV E6/E7 mRNA, and 7 genital infections–*C*. *trachomatis*, *N*. *gonorrhea*, *T*. *vaginalis*, *M*. *hominis*, *M*. *genitalium*, *U*. *urealyticum*, *U*. *parvum*. Results showed that women with all grades of cervical intraepithelial neoplasia (CIN) more often were smokers, had increased vaginal pH levels, and had positive HR-HPV DNA and HR HPV E6/E7 mRNA expression. Abnormal vaginal microflora, especially types associated with aerobic vaginitis, and *M*. *hominis* were significantly more often found in women with CIN2+. The presence *of C*.*trachomatis*, *U*. *parvum*, *and U*.*urealyticum* did not differ between the groups. The most important factors independently associated with CIN2+ were positive high-risk HPV E6/E7 mRNA expression (OR 59.4, 95% CI 14.84–237.51), and positive high-risk HPV DNA (OR 3.9, 95% CI 1.16–13.23). Higher education level was associated with reduced risk of CIN2+ (OR 0.2, 95% CI 0.07–0.71). In conclusion, this study reports HR-HPV DNA of the most common six types and E6/E7 mRNA positivity as the most significant factors associated with CIN2+ lesions and higher education related to lower risk of high-grade cervical lesions.

## Introduction

Cervical cancer is the second most common malignant disease in women of reproductive age worldwide, and despite the existing screening programs, it is still a significant healthcare burden [1]. In the last decades, the cervical cancer screening program has evolved. Many countries have switched from less precise cytology testing to more sensitive high-risk human papillomavirus (HR-HPV) detection. However, HPV infection is highly prevalent and is the most common sexually transmitted infection, but only a small percentage of infected women subsequently develop a cervical malignant disease [2]. It is already known that HR-HPV infection is necessary but not the only factor needed to develop cervical cancer [3]. Up to 90% of HPV infections are transient and clear spontaneously within 1–2 years [4]. Only about 10% persist and progress to a transforming HPV infection that can lead to the development of cervical cancer [4, 5]. For that reason, it is crucial to estimate cervical cancer development risk in the population of HR-HPV-positive women and to avoid unnecessary examinations and treatment in low-risk individuals.

The development of artificial intelligence and machine learning is allowing to make risk-prediction models for better cancer prediction [6–9]. However, there are many factors which can influence the course of HR-HPV infection. For example, multiple sexual partners, smoking, use of oral contraceptives, and some sexually transmitted infections increase the risk of HR-HPV persistence and progression to high-grade cervical intraepithelial neoplasia [10, 11]. The expression of HR-HPV E6/E7 mRNA has also shown great value in predicting the course of the CIN [12–14].

In recent years the role of vaginal microenvironment in the development of cervical cancer also has been studied extensively. But still, the results are controversial. It looks that higher microbial diversity and the lack of lactobacilli are associated with cervical lesions, but the role of certain infections remains unclear [3, 15–17]. We have previously reported the importance of aerobic vaginitis in association with CIN [18]. There is increasing demand to identify different risk factors for cervical cancer development, which can be included in risk-prediction models.

The current study aimed to identify associations between different personal factors, vaginal microflora, and sexually transmitted, including HR HPV infection and different degrees of cervical precancerous changes.

## Material and methods

The study was conducted from the 1^st^ of January 2016 until the 31^st^ of December 2020. Data for research purposes were accessed from the 1^st^ of February to the 31^st^ of October 2023. 112 patients with abnormal cervical cytology results referred for colposcopic examination to Riga East University Hospital were selected as a study group. The control group consisted of 120 women who came for a routine gynecological examination. The exclusion criteria were: age under 18 years old, pregnancy, and personal history of cervical precancerous/malignant disease. The study was conducted by the Declaration of Helsinki and approved by the Ethical Committee of Riga Stradins University (Ethical approval code 39/24). All participants signed an informed consent.

All patients included in the study had interviews, gynecological examinations, and colposcopies performed by certified colposcopy specialists (JZ, IJ, KP). The custom-designed questionnaire with questions about different personal factors (age, education level, smoking, marital status, and type of contraception) was filled out during the interview. Low education level was considered if women had primary (duration of education 9 years) or secondary education (duration of education 12 years), but high education level if at least a bachelor’s degree had been obtained. Contraception methods were defined as “effective method” (combined hormonal contraceptives, progestin-only contraception, intrauterine device, hormonal intrauterine system, male/female sterilization), “condom”, “withdrawal” (interrupted intercourse), and “no contraception”.

An unmoistened vaginal speculum was used to perform gynecological examination. The material from the upper vaginal fornix was taken for the pH measurement and wet mount microscopy. Vaginal pH was measured with Machery-Nagel pH strips with the range of 3.1–7 [19]. pH >4.4 was considered abnormal. For wet-mount microscopy, the material was spread on the glass slide, air-dried, and later rehydrated with a drop of normal saline [20]. Microscopic examinations included the evaluation of lactobacillary grades (LBG), the number of leucocytes, the proportion of toxic leucocytes, the presence of ‘clue’ cells, the proportion of parabasal cells, and background flora [21]. LBG were divided according to the proportion between lactobacillus and other bacteria (Donders’ modification of Schröder’s classification [22]): LBG I—the dominant presence of lactobacillus morphotypes, no other bacteria; LBG IIa—lactobacilli dominance, but other bacteria present; LBG IIb—other microorganisms outnumbering lactobacilli; LBG III—no lactobacilli, other bacteria present. LBG III was further divided into three subgroups: bacterial vaginosis (BV), aerobic vaginitis (AV), and mixed BV-AV flora. Normal vaginal microbiota was defined as LBG I and IIa, but the abnormal as LBG IIb and III. A predominant granular microflora with uncountable bacteria all over the slide and >20% of ‘clue cells’ was defined as full-blown BV, while mixed areas with streaks of BV-like microflora or sporadic ‘clue cells’ combined with other types of microflora were classified as partial BV [23]. The severity of AV was assessed using the AV score, described by Donders [21]. The score parameters were: LBG, the number of leucocytes, the proportion of toxic leucocytes, background microflora, and the proportion of parabasal epitheliocytes. A composite AV score <3 represented no AV, the score 3–4—light AV, 5–6—moderate AV, and >6—severe AV. To evaluate the effect of a definite abnormal microbiota type on the development of CIN, we divided pathological microbiota as follows: ‘any AV flora’ included LBG III AV, mixed AV-BV, and LBG IIb with signs of AV; and ‘any BV’ included LBG III BV, mixed AV-BV and LBG IIB with signs of BV (partial BV). We have also assessed the severity grades of AV as a risk factor for CIN. Moderate to severe AV (msAV) was defined as an AV score of 5 or more.

To determine the presence of DNA for the six most common high-risk HPV types (16/18, 31, 33, 45, 58) within the test sample from the cervix, two approaches were used. First, the MICROLAB Nimbus IVD (HAMILTON) device was used for HPV DNA detection within the sample, and the universal nucleic acid extraction kit STARMag 96 X 4 Universal Cartridge Kit (Seegene, CAT. No. 7444300.4.UC384) was used for the extraction procedure. Then, a multiplex real-time PCR assay was carried out using Anyplex (TM) II HPV HR Detection assay (Seegene, CAT. No. HP7E00X). Afterward, E6/E7 viral messenger RNA (mRNA) from 14 high-risk types of HPV (16, 18, 31, 33, 35, 39, 45, 51, 52, 56, 58, 59, 66, 68) was determined by using automated hybridization and amplification system that includes fully automated sample-to-answer instrument Panther System (Hologic), Panther Run Kit (Hologic, CAT. No. 303096) and the Aptima HPV assay (Hologic, CAT. No. 303093).

The presence of seven genital infections (*Chlamydia trachomatis*, *Neisseria gonorrhoeae*, *Trichomonas vaginalis*, *Mycoplasma hominis*, *Mycoplasma genitalium*, *Ureaplasma urealyticum*, *Ureaplasma parvum*) was determined by molecular diagnostic methods: DNA extraction and real-time polymerase chain reaction. Swab samples were obtained from the cervix and preserved in *MSwab®* (COPAN, CAT. No. 6E011N) transport media. DNA extraction was carried out using the automated liquid handling instrument *MICROLAB Nimbus IVD* (HAMILTON) and universal nucleic acid extraction kit STARMag 96 X 4 Universal Cartridge Kit (Seegene, CAT. No. 7444300.4.UC384), where the extraction procedure is based on reversible absorption of nucleic acids to paramagnetic beads under appropriate buffer conditions. Afterward, the extracted DNA was amplified by using multiplex real-time PCR assay developed using the proprietary DPO™ and TOCE™ technologies *AnyPlex*^TM^ II *STI*-*7e* panel assay (Seegene, CAT. No. SD7701X) and real-time PCR device *CFX 96 C1000* (Bio-Rad Laboratories, Inc.).

All study participants underwent colposcopy examinations according to local and European colposcopy guidelines. From all patients in the study group (referred due to abnormal cytology) and in case of visual suspicion of cervical pathology in the control group patients, at least two biopsies were taken. A histological examination was performed at the Pathology Center of Riga East University Hospital. The results were classified as negative, CIN1, CIN2, CIN3, and carcinoma. Cases with CIN1-2 or CIN2-3 lesions were upgraded and included correspondingly in the group of individuals with CIN2 or CIN3. In the control group, if colposcopy was adequate and there were no signs of precancerous lesions, this was interpreted as “no CIN”. We combined all CIN severity groups during data analysis and analyzed them as “all CIN”. CIN2, CIN3, and carcinoma cases were also analyzed together as CIN2+. We have compared results between “no CIN”, CIN1, CIN2+, and “all CIN” groups.

Statistical analysis was performed with Microsoft Excel 2020 and IBM SPSS 20.0. A t-test was used to compare the mean ages between the groups. Pearson chi-square or Fisher’s exact test was used to assess the relations between variables. A p-value <0.05 was considered statistically significant. Univariate and multivariate logistic regression was used to determine the association between CIN2+ and different risk factors. The multiple logistic regression included variables that showed a significant association in the univariate analysis (p<0.05). The risk of CIN2+ development, depending on various risk factors, was calculated as odds ratios.

## Results

After histological examination, two study group patients were diagnosed with benign cervical lesions (cervicitis) and, therefore, were added to the “no CIN” group. Five cases from the control group were excluded from the study (one due to an unreadable microscopy slide and four because of an incomplete questionnaire). So, as a result, the final analysis included 110 cases with CIN and 117 “no CIN” cases. There were 31 (28.2%) women with CIN1, 57 (51.8%) with CIN2, 21 (19.1%)—CIN3, and 1 (0.9%) cervical cancer patient in the study group.

The mean age of the “all CIN” women was 35.0 (±9.3) years, and in the “no CIN” group 37.1 (±8.0) years (p = 0.059). In the age group below 30 CIN1, and “all CIN” were more common than “no CIN” (p = 0.032 and p = 0.039, respectively). Other differences between age groups and CIN severity were not observed. There were no statistically significant differences between the groups in terms of marital status and contraception methods (Table 1).

**Table 1:** Characteristics of the groups.

Factor	no CIN n = 117 n(%)	CIN1 n = 31 n(%)	CIN2+ n = 79 n(%)	all CIN n = 110 n(%)	P value noCIN vs. allCIN	P value noCIN vs CIN1	P value noCIN vs CIN2+	P value CIN 1 vs CIN2+
Mean age	37.1 (±8.0)	34.4 ± 1.7	35.5 ± 1.0	35.0 (±9.3)	0.059	0.103	0.118	0.684
Age group								
≤30	27 (23.1)	13 (41.9)	26 (32.9)	39 (35.5)	0.032	0.039	0.093	0.661
31–49	87 (74.4)	16 (51.6)	48 (60.8)	64 (58.2)				
≥50	3 (2.6)	2 (6.5)	5 (6.3)	7 (6.3)				
Education level								
lower	25 (21.4)	15 (48.4)	37 (46.9)	52 (47.3)	<0.0001	0.003	<0.0001	0.083
higher	92 (78.6)	16 (51.6)	42 (53.1)	58 (52.7)				
Relationship								
Registered marriage	60 (51.3)	18 (58.0)	43 (54.4)	61 (55.5)	0.785	0.877	0.847	0.950
Non-registered marriage	44 (37.6)	10 (32.3)	29 (36.7)	39 (35.5)				
Lonely	13 (11.1)	3 (9.7)	7 (8.9)	10 (9.0)				
Contraception group								
Effective	29 (24.8)	7 (22.6)	22 (27.8)	29 (26.4)	0.344	0.287	0.628	0.702
Condoms	40 (34.2)	6 (19.4)	21 (26.6)	27 (24.5)				
Withdrawal	8 (6.8)	2 (6.4)	4 (5.1)	6 (5.5)				
No contraception	40 (34.2)	16 (51.6)	32 (40.5)	48 (43.6)				
Smoking								
No	106 (90.6)	19 (61.3)	53 (67.1)	72 (65.5)	<0.0001	<0.0001	<0.0001	0.565
Yes	11 (9.4)	12 (38.7)	26 (32.9)	38 (34.5)				
HR-HPV DNA of six most common types								
Negative	108 (92.3)	15 (48.4)	28 (35.4)	43 (39.1)	<0.0001	<0.0001	<0.0001	0.211
Positive	9 (7.7)	16 (51.6)	51 (64.6)	67 (60.9)				
HR-HPV E6/E7 mRNA								
Negative	103 (88)	13 (41.9)	8 (10.1)	21 (19.1)	<0.0001	<0.0001	<0.0001	<0.0001
Positive	14 (12)	18 (58.1)	71 (89.9)	89 (80.9)				
pH								
≤ 4.4	95 (81.2)	18 (58.1)	39 (49.4)	57 (51.8)	<0.0001	0.007	<0.0001	0.411
>4.4	22 (18.8)	13 (41.9)	40 (50.6)	53 (48.2)				
*Chlamydia trachomatis*	4 (3.4)	3 (9.7)	3 (3.8)	6 (5.4)	0.529	0.160	1.000	0.348
*Neisseria gonorrhoea*	0	0	0	0	-	-	-	-
*Trichomonas vaginalis*	0	0	2 (2.5)	2 (1.8)	0.234	-	0.161	1.000
*Mycoplasma genitalium*	0	0	1 (1.3)	1 (0.9)	0.485	-	0.403	1.000
All STI	4 (3.4)	3 (9.7)	6 (7.6)	9 (8.2)	0.157	0.160	0.206	0.710
*M*.*hominis*	2 (1.7)	3 (9.7)	7 (8.9)	10 (9.1)	0.016	0.062	0.032	1.000
*U*.*parvum*	35 (29.9)	13 (41.9)	29 (36.7)	42 (38.2)	0.189	0.204	0.320	0.612
*U*.*urealyticum*	8 (6.8)	1 (3.2)	9 (11.4)	10 (9.1)	0.530	0.685	0.266	0.277
Non STI *Mycoplasmas*	41 (35.0)	15 (48.4)	36 (45.6)	51 (46.4)	0.083	0.173	0.139	0.790
Microflora								
Normal	76 (65.0)	17 (54.8)	38 (48.1)	55 (50.0)	0.023	0.300	0.019	0.525
Abnormal	41 (35.0)	14 (45.2)	41 (51.9)	55 (50.0)				
Any BV	9 (7.7)	8 (25.8)	8 (10.1)	16 (14.5)	0.099	0.010	0.553	0.036
Any AV	24 (20.5)	6 (19.4)	29 (36.7)	35 (31.8)	0.052	0.887	0.012	0.079
msAV	6 (5.1)	2 (6.5)	13 (16.5)	15 (13.6)	0.038	0.674	0.009	0.225

Women with all grades of CIN more often were smokers, had increased vaginal pH levels, and had positive HR-HPV DNA and HR HPV E6/E7 mRNA expression compared to those without CIN (Table 1), but women without CIN more frequently had higher education compared to cases with CIN.

Positive *Chlamydia trachomatis* cases were almost equally often found in the study and the control group. We had no cases of *N*.*gonorrhoea*. The two cases of *Trichomonas vaginalis* and one *Mycoplasma genitalium* were diagnosed in CIN2+ patients. *Mycoplasma hominis* was more common in CIN 2+ (7/79, 8.9%; OR 5.6, 95% CI 1.1–27.7) group compared to healthy women (2/117, 1.7%, p = 0.032), but *Ureaplasma parvum* and *U*.*urealyticum* did not differ between the groups (Tables 1 and 2).

**Table 2:** Univariate logistic regression CIN2+ compared to no CIN.

	Odds ratio	95% Confidence Interval	p-value
High education level	0.3	0.17–0.58	<0.0001
Smoking	4.7	2.2–10.3	<0.0001
Positive HR-HPV DNA of six most common types	21.9	9.6–49.7	<0.0001
Positive HR HPV E6/E7 mRNA expression	65.3	26.0–163.8	<0.0001
pH	4.4	2.3–8.4	<0.0001
*M*. *hominis*	5.6	1.1–27.7	0.035
Abnormal microflora	2.0	1.1–3.6	0.020
anyAV	2.2	1.2–4.3	0.013
msAV	3.6	1.3–10.0	0.012

Smoking (OR 4.7, 95% CI 2.2–10.3), abnormal vaginal microflora (OR 2.0, 95% CI 1.1–3.6), any AV microflora changes (OR 2.2, 95% CI 1.2–4.3), and msAV (OR 3.6, 95% CI 1.3–10.0) were significantly more often found in women with CIN2+ compared to those without CIN. In turn, BV was associated with CIN1 and was observed less in healthy and CIN2+ women. On the contrary, high education level showed a protective effect and was more often found in healthy women (OR 0.3, 95% CI 0.17–0.58) (Tables 1 and 2).

Multivariate logistic regression analysis revealed that the most important factors independently associated with CIN2+ were positive HR-HPV E6/E7 mRNA expression (OR 59.4, 95% CI 14.84-

237.51), positive HR-HPV DNA (OR 3.9, 95% CI 1.16–13.23). Higher education level was associated with reduced risk of CIN2+ (OR 0.2, 95% CI 0.07–0.71) (Table 3).

**Table 3:** Multivariate logistic regression CIN2+ compared to no CIN.

	Odds ratio	95% CI	p-value
High education level	0.2	0.07–0.71	0.012
Smoking	1.7	0.46–6.49	0.414
Positive HR-HPV DNA status of six most common types	3.9	1.16–13.23	0.028
Positive HR-HPV E6/E7 mRNA expression	59.4	14.84–237.51	<0.0001
pH	2.2	0.66–7.35	0.199
*M*.*hominis*	1.66	0.08–32.09	0.776
Abnormal microflora	1.3	0.31–5.41	0.723
anyAV	0.73	0.15–3.57	0.696
msAV	3.4	0.37–31.31	0.283

## Discussion

In this study, we have found that positive HR-HPV DNA of the most common types and E6/E7 mRNA are the most important factors associated with CIN2+, in turn, higher education showed a protective effect.

Cervical cancer rarely develops without HR-HPV [24]. Therefore, it is clear that the detection of HR-HPV DNA is common in high-grade cervical lesions [25]. This study demonstrated an almost 4-fold increased risk of CIN2+ in HR-HPV DNA-positive individuals. European guidelines for quality assurance in cervical cancer screening recommend HR-HPV DNA detection as primary screening for women older than 30 [26]. This strategy has already been implemented by many European countries [27]. Interestingly, in our study, one-third of CIN2+ cases were HR-HPV DNA-negative. This could be mainly related to the fact that only the six most common HR-HPV types were analyzed, although these six genotypes (16, 18, 31, 33, 45, 58) contribute to more than 90% of invasive cervical cancer cases [28]. Another explanation may be the fact that low-risk HPV can induce the development of CIN1-2 lesions [29]. In the “no CIN” group, almost 8% were HR-HPV DNA positive. These could be women with either newly acquired infection or those in whom cell transformation has not yet occurred. Other studies show that 5.1–29.3% of cytologically normal smears could be HR-HPV positive depending on the population and geographical region [29].

HPV is a widespread infection and is self-limiting in most cases [2], so it is crucial to identify those HR-HPV-positive women who are at risk of subsequently developing high-grade cervical neoplasia. It is well established that the expression of E6 and E7 oncogenes inactivates the tumor-suppressing proteins p53 and pRB in the host cell and promotes malignant transformation [30–33]. Therefore, the detection of E6/E7 mRNA expression has great potential to be used as a biomarker to distinguish between transient and transforming HR-HPV infection. In recent years, many studies have shown the diagnostic value of this test [12–14, 30, 34]. E6/E7 mRNA detection has the potential to be used as a triage or primary HPV screening test. It has shown very high sensitivity [35, 36], and particularly works well in triaging ASCUS patients [35, 37]. The current study demonstrated that being positive for HR-HPV E6/E7 mRNA was significantly associated with CIN2+ lesions and showed the highest odds ratio.

Education level was the third factor that showed the most importance in association with CIN2+. Women without CIN more often had higher education levels. Previous studies have shown that lower education is a significant risk factor for cervical neoplasia–women with lower education levels more often had CIN of all grades [38–40]. On one hand, low education in general is related to poor health knowledge–women do not know about cervical cancer risk factors and symptoms [41], on the other hand, poorly educated women could have high-risk sexual behavior (young age at first intercourse, number of sexual partners, lack of contraception use) [42]–the leading risk factor for HPV infection [10, 11]. Unfortunately, our study did not address questions about sexual behavior. Society education could improve knowledge about HPV infection, its risk factors, and available prophylaxis methods. Another marker of education level could be the knowledge about sexual health. Although we have not asked direct questions about this topic, we could make some assumptions based on the answers about the contraception method. Albeit statistically significant differences between the groups were not observed, it looks quite clear that women in the “all CIN” group less frequently use condoms and mostly don’t use any method at all.

Smoking was also more commonly found in women with cervical precancerous lesions. Smoking is a well-known cervical cancer risk factor. It affects humoral and cellular immunity in the vagina and cervix, and lowers the ability of the immune system to clear from HPV infection, and therefore could promote persistency and carcinogenesis [43–46]. However, smoking could also be an indirect indicator of the education level.

Vaginal pH level and wet mount microscopy are fast and almost low-cost point-of-care tests to evaluate vaginal microflora [21, 47]. They have similar diagnostic value but are much more affordable and convenient in daily clinical practice than expensive and time-consuming molecular biology techniques [18, 48, 49]. In this study, we reported an association between increased vaginal pH and abnormal vaginal microflora with CIN2+, which is in line with other studies about altered vaginal microecology [3, 15–17, 50–52]. We have previously reported the importance of aerobic vaginitis, especially in its moderate to severe form, in association with CIN2+ lesions. It should not be ignored when analyzing the vaginal microenvironment [18]. A newer publication has supported our suggestion to pay greater attention to AV [53]. On the contrary, BV was found more often in CIN1 cases and was not associated with high-grade cervical lesions. This could be explained by the fact that both BV and low-grade lesions induced by transient HPV infection could be more related to sexual activity [18, 54, 55].

The vaginal microenvironment plays a significant role in the development of cervical pathology. However, the exact mechanism is not yet evident.

The most prevalent sexually transmitted infection in our study population was *Chlamydia trachomatis*, but we were not able to find any significant differences between the groups. Association between *C*.*trachomatis* infection and cervical lesions has been demonstrated in the literature [56–58], but we were not able to repeat these results. There was a trend for more positive cases in the CIN1 group. This could be explained by the fact that CIN1 is mainly associated with the transient HPV infection, and sexual behavior is the most important risk factor for HR-HPV infection [55].

The only cases of *T*.*vaginalis* and *M*.*genitalium* were found in the CIN2+ group, but the number is too small to make any correlations. The meta-analysis on the association between *T*.*vaginalis* infection and the risk of cervical cancer has concluded that infected individuals have a greater risk of cervical cancer [59]. In turn, data about *M*.*genitalium* are not so clear. Most of the studies about *Mycoplasmataceae* are focused on the role of *M*.*hominis*, *U*.*parvum*, and *U*.*urealyticum* in the development of cervical neoplasia. However, there is data on *M*.*genitalium* as a significant CIN risk factor [60]. A meta-analysis published in 2018 reveals an association between *M*.*genitalium* and high-risk HPV infection, but not with cervical cytopathology [61].

On the other hand, it has been reported that *M*.*hominis*, *U*.*parvum*, and *U*.*urealyticum* could be significant risk factors for cervical dysplasia [61]. Our results showed a significant association between *M*.*hominis* and CIN2+. In the univariate analysis, *M*.*hominis* infection was the third most important CIN2+ risk factor with more than a 5-fold increase, but the significance of that finding was lost in the multivariate analysis. The possible role of genital Mycoplasmas in the development of cervical lesions is extensively discussed in the literature. Many publications are reporting significant associations between different *Mycoplasmas* and high-risk HPV infection [53, 61, 62], but their role in promoting carcinogenesis is not clear yet. In the very recent publication by *Disi et al*., it was suggested that different *Mycoplasma* subtypes are differently associated with the HPV infection, and therefore, detailed *Mycoplasma* typing could be significant in clinical practice [53].

The significance of many other factors influencing the course of cervical cancer development is still disputable. The chance of getting cancer after an HPV infection is low [63]. However, it is of great importance to identify those relatively rare cases of high-grade lesions to treat them and thereby prevent the development of cervical cancer. Especially taking into account that these are women of reproductive age [64, 65]. Since HPV-based screening is favorable due to its higher sensitivity [66], there is a need to distinguish those few HPV-positive individuals at high risk of developing cervical cancer. So many factors are nowadays known to affect the course of HPV infection [3, 10, 11]. It could be that a true combination of these factors matters. It looks like we need some risk prediction models combining these factors. The development of such predicting tools has already started using artificial intelligence. Models including HR-HPV type, smoking, sexual behavior, demographic, and genetic factors have already been tested [6–8, 67, 68]. Considering possible geographical and ethnic variations between populations in terms of HR-HPV types, vaginal and sexually transmitted infections, and sexual behavior, data from all around the world would be needed for correct machine learning and developing a universal risk calculation tool.

The strength of this study is histologically proven CIN grades and simultaneous analysis of different personal factors, HR-HPV DNA and mRNA, vaginal environment, and sexually transmitted infections. Unfortunately, we represent a relatively small number of participants and analyzed only six most common HR-HPV genotypes, which is a major limitation of our study and could influence its statistical power.

In conclusion, this study reports HR-HPV DNA of the most common six types and E6/E7 mRNA positivity as the most significant factors associated with CIN2+ lesions and higher education related to lower risk of high-grade cervical lesions. To our knowledge, this is one the first studies that analyzed many different factors–behavioral, social, related to vaginal environment and HR-HPV infection–at the same time. Although the statistical power when evaluating some individual risk factors (smoking, increased vaginal pH, and altered vaginal microenvironment) for cervical disease was lost, it is still important to take them into account. Those factors should be considered for developing powerful machine learning-based individual risk prediction programs for personalized cervical cancer prevention.
